# Effects of antiplatelet therapy on menstrual blood loss in reproductive-aged women: a systematic review

**DOI:** 10.1016/j.rpth.2023.102295

**Published:** 2023-12-09

**Authors:** Eva K. Kempers, Johanna A. van der Zande, Paula M. Janssen, Jérôme M.J. Cornette, Jolien W. Roos-Hesselink, Marieke J.H.A. Kruip

**Affiliations:** 1 Department of Hematology, Erasmus University Medical Center, Rotterdam, the Netherlands; 2 Department of Cardiology, Erasmus University Medical Center, Rotterdam, the Netherlands; 3 Department of Obstetrics and Gynecology, Erasmus University Medical Center, Rotterdam, the Netherlands; 4 Department of Neurology, Erasmus University Medical Center, Rotterdam, the Netherlands

**Keywords:** aspirin, clopidogrel, menorrhagia, platelet aggregation inhibitors, systematic review

## Abstract

**Background:**

The effects of antiplatelet therapy on menstrual bleeding have not been well characterized.

**Objectives:**

To systematically review the effects of antiplatelet therapy on menstrual bleeding.

**Methods:**

A literature search was performed for studies of reproductive-aged women who received antiplatelet therapy. Characteristics of menstrual bleeding both before and after initiation of antiplatelet therapy and from comparison groups were collected. Two reviewers independently assessed the risk of bias in individual studies.

**Results:**

Thirteen studies with a total of 611 women who received antiplatelet therapy were included. Types of antiplatelet drugs used were aspirin (*n* = 8), aspirin and/or clopidogrel (*n* = 2), prasugrel (*n* = 1), and not specified (*n* = 2). Risk of bias was assessed at moderate (*n* = 1), serious (*n* = 8), critical (*n* = 2), and no information (*n* = 2). Three studies reported changes in menstrual blood loss volume. One of these showed no increase during antiplatelet therapy; the other 2 studies suggested that aspirin may increase menstrual blood loss volume. In 3 studies that assessed the duration of menstrual bleeding, up to 13% of women reported an increased duration of menstruation. In 5 studies that reported the intensity of menstrual flow, 13% to 38% of women experienced an increase in the intensity of flow. Five studies reported the prevalence of heavy menstrual bleeding in women who received antiplatelet therapy, with estimates ranging from 7% to 38%.

**Conclusion:**

There is lack of high-quality data on the effects of antiplatelet therapy on menstrual bleeding. Aspirin may increase menstrual blood loss, at least in a minority of women, whereas the effects of P2Y12 inhibitors are unknown.

## Essentials

• Antiplatelet therapy is used for prevention and treatment of cardiovascular disease.
• We performed a systematic review on the effects of antiplatelet therapy on menstrual blood loss.
• We identified 13 studies in total, the majority at high risk of bias.
• Low-dose aspirin may increase menstrual blood loss, at least in a minority of women.

## 1 Introduction

There is a growing burden of cardiovascular disease among women of reproductive age, which is associated with increased use of antiplatelet therapy for the purpose of secondary prevention [[1], [2], [3]]. While it is unclear for antiplatelet therapy, anticoagulant therapy, such as direct oral anticoagulants (DOACs) and vitamin K antagonists (VKAs), in women with an active menstrual cycle has been associated with abnormal uterine bleeding (AUB). The incidence of new-onset AUB among women initiating anticoagulant therapy is estimated at 60% [4]. AUB is an overarching term that refers to menstrual bleeding that is abnormal in frequency, duration, or volume [5]. The latter is also referred to as heavy menstrual bleeding (HMB) or previously referred to as menorrhagia, which is defined as “excessive menstrual blood loss that interferes with the patient’s physical, emotional, social, and/or material quality of life” [6,7]. An alternative, more strict definition of HMB, which requires the direct measurement of menstrual blood loss, is 80 mL or more menstrual blood loss per cycle [8,9]. The prevalence of HMB in the general population of reproductive-aged women is estimated between 10% and 30%, depending on whether HMB was defined based on objectively measured menstrual blood loss or self-reported measures [10]. Menstrual blood loss can be assessed with the alkaline hematin technique, which is considered the gold standard and most objective method [11]. Due to its practical limitations, other assessment methods, such as the pictorial blood loss assessment chart (PBAC), are more frequently used in clinical studies [12]. The PBAC is a self-administered, semiquantitative tool to assess menstrual blood loss, and a score of 100 is considered indicative of 80 mL menstrual blood loss [13].

HMB negatively affects quality of life, labor productivity and could result in iron deficiency and anemia [10,14,15]. Moreover, these adverse effects could also negatively affect adherence to anticoagulant therapy, as has been suggested by studies on DOACs [16,17]. However, the effects of platelet aggregation inhibitors on menstrual blood loss have not been well characterized. Awareness of the possible consequences of antiplatelet therapy on menstrual blood loss is lacking among prescribers, and physicians often do not ask about complaints related to menstrual blood loss [18]. Nevertheless, the possible adverse effects of antiplatelet therapy on menstrual blood loss could represent an important issue for premenopausal women. Therefore, the objectives of this systematic review were to examine the effects of antiplatelet therapy on menstrual blood loss in reproductive-aged women.

## 2 Methods

This systematic review is reported in accordance with the Preferred Reporting Items for Systematic Reviews and Meta-Analyses statement [19]. The review protocol was registered at PROSPERO (CRD42023388166).

### 2.1 Search strategy

The following databases were searched from inception until November 28, 2022: Ovid MEDLINE, Embase, Web of Science Core Collection, Cochrane Central Register of Controlled Trials, and Google Scholar. The full search strategy, which was developed by a biomedical information specialist, is displayed in the Supplementary search strategy.

### 2.2 Study selection

Titles and abstracts of each record were screened independently by 2 reviewers (E.K.K. and J.A.v.d.Z.), followed by full-text screening of the articles that were considered to be eligible. In case of discrepancies, consensus was reached through discussion. If necessary, a third reviewer was consulted. Full text of conference abstracts that were considered eligible was retrieved, if possible. Studies were included if the study population included adolescent and/or adult women of reproductive age who did or did not use contraceptives of any kind. Study participants had to be treated with platelet aggregation inhibitors of any kind, including dual and triple antiplatelet therapy. Menstrual blood loss had to be assessed with the (modified) PBAC, the (modified) menstrual pictogram, the alkaline hematin technique, menstrual fluid loss, counts of sanitary items, measurement of iron or labeled red blood cells in pads, duration of menstruation, questionnaires, or self-perception methods. In addition, studies had to be written in English, and the population at risk had to be identifiable (ie, the proportion of reproductive-aged female study participants).

Studies were excluded if all participants were pregnant, if all participants used anticoagulants only or combined with antiplatelet drugs, or if all participants had a bleeding disorder. The latter included inherited or acquired von Willebrand disease, carriership of hemophilia, and inherited but not acquired platelet disorders. Moreover, animal studies, case reports, nonsystematic reviews, and conference abstracts of which the full text could not be retrieved were excluded.

### 2.3 Data collection

Two reviewers (E.K.K. and J.A.v.d.Z.) independently collected data from eligible studies. Disagreements were resolved through discussion. Any measure of menstrual blood loss, both before and after initiation of antiplatelet therapy, was extracted, as well as measures of menstrual blood loss from comparison groups if reported. Individual studies were allowed to report data on multiple aspects of menstrual bleeding, such as intensity and duration of menstrual blood loss, with the use of several methods. In addition, single studies were allowed to report on menstrual bleeding at multiple time points during follow-up. Eligible measures were categorized into 1 of 4 groups: menstrual blood loss volume, duration of menstrual bleeding, intensity of menstrual flow, or incidence and prevalence of HMB. The latter was defined as any estimate of the occurrence of HMB during study follow-up, according to the study authors’ definition. Other variables on which data were collected from eligible studies were study characteristics (year of publication, country, study design, and sample size); participant characteristics; characteristics of the antiplatelet drug used, such as type, dose, and duration; the use of contraceptives, such as oral contraceptives or intrauterine devices (IUDs); and concomitant use of nonsteroidal anti-inflammatory drugs.

### 2.4 Risk of bias assessment

Risk of bias in individual studies was assessed independently by the 2 reviewers according to the Risk of Bias in Non-randomised Studies - of Interventions assessment tool [20]. The risk of bias was assessed across 7 domains based on the concept of target trial emulation. Disagreements between reviewers were resolved through discussion, and if necessary, a third reviewer (M.J.H.A.K) was consulted. Both domain-level risk of bias as well as overall risk of bias judgments were reported.

### 2.5 Sex and gender

The term women in this study refers to individuals of female sex. People who menstruate may identify either with gender or as nonbinary.

## 3 Results

### 3.1 Study selection

**Figure 1:**
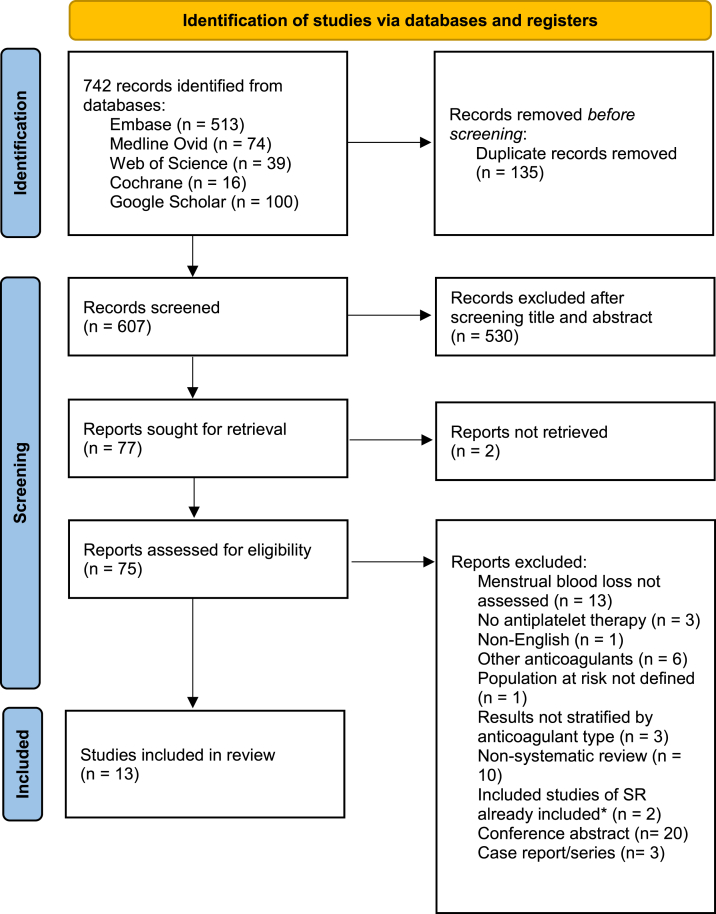
Flow diagram of the study selection process. ∗Systematic review (SR) of the studies that were already included. This figure was adapted from the Preferred Reporting Items for Systematic Reviews and Meta-Analyses 2020 flow diagram [21].

In total, 742 records were identified by our database search, of which 607 potentially relevant unique articles were screened based on titles and abstracts (Figure 1) [21]. Full-text screening was subsequently performed on 75 records, of which 13 studies were finally included [[22], [23], [24], [25], [26], [27], [28], [29], [30], [31], [32], [33], [34]]. Excluded studies and reasons for exclusion are listed in the Supplementary material (Item 16b of PRISMA 2020 checklist).

### 3.2 Study characteristics

Thirteen studies with a total of 611 women who used any type of antiplatelet drug were included [[22], [23], [24], [25], [26], [27], [28], [29], [30], [31], [32], [33], [34]] (Table 1). Seven studies had an experimental design: 3 (randomized) double-blind crossover studies [[22], [23], [24]], 3 randomized (double-blind) controlled trials [26,27,31], and 1 prospective study without control group [25]. The other studies had an observational design: 3 prospective cohort studies [28,30,34], 2 retrospective cohort studies [29,32], and 1 cross-sectional study [33]. Types of antiplatelet drugs used were aspirin only [[22], [23], [24], [25],[27], [28], [29],^31^], aspirin and/or clopidogrel [30,33], prasugrel [26], and unspecified antiplatelet drugs [32,34]. Doses of aspirin ranged between 75 and 1000 mg. In 4 studies, the use of the antiplatelet drug (aspirin) was intended as a therapy for complaints of increased menstrual blood loss related to copper-containing IUD use or for dysmenorrhea, and its use was restricted to the period of menstruation only [[22], [23], [24], [25]]. The doses of aspirin in these studies were higher, ranging from 500 to 1000 mg, multiple times a day. Contraceptive use was reported in 8 studies: in 3 studies, participants did not use contraceptives [24,28,30]; in 2 studies, some or all participants had an IUD [23,25]; in 2 studies, some participants used oral contraceptives [27,33]; and in 1 study, some participants used oral contraceptives or had an IUD [22]. Table 2 summarizes the characteristics of participants of the included studies. Five studies included regularly cycling women only [[22], [23], [24], [25],^28^]. Others included different patient populations, such as patients with sickle cell disease [26], patients with antiphospholipid antibodies and/or systemic lupus erythematosus [27,29], patients who had venous thromboembolism (VTE) [31], or patients with Fontan circulation [32,34].

**Table 1:** Characteristics of included studies.

Author, year	Study design	Country	Sample size^a^	Antiplatelet drug type	Antiplatelet drug dose	Age^b^	Contraceptive use	Concomitant use of NSAIDs
Corson and Bolognese [22], 1978	Randomized double-blind crossover study	United States	33	Aspirin	Aspirin 325 mg (2 tablets every 4 h as necessary for pain relief)	Mean (range), 24.7 (14-48)	Oral contraceptives: 5 IUD: 4	Not reported
Hahn and Petruson [23], 1979	Nonrandomized double-blind crossover study	Sweden	33	Aspirin	Aspirin 500 mg (starting on the first day of menstruation, 1 tablet 3 times a day, continued during the bleeding period)	Not specified	Oral contraceptives: none IUD copper-containing: 10	Not reported
Krishna et al. [24], 1980	Nonrandomized double-blind crossover study	India	39	Aspirin	Aspirin 300 mg (2 tablets at onset of dysmenorrhea, continued at 8-h intervals until symptoms disappeared)	Mean (range), 20 (14-26)	Oral contraceptives: none IUD: not reported	Not used
Pedron et al. [25], 1987	Prospective intervention study	Mexico	53	Aspirin	Aspirin 500 mg (2 tablets every 8 h from the onset of menstrual bleeding for 5 d)	Not specified	Oral contraceptives: none IUD: all	Not reported
Wun et al. [26], 2013	Randomized double-blind controlled phase 2 trial	United States, Canada	21	Prasugrel	Prasugrel 5 mg daily	Mean, 32.9 (not reported separately for female participants)	Not reported	Not used
Cuadrado et al. [27], 2014	Randomized open-label controlled trial	United Kingdom, Europe, Mexico	80	Aspirin	Aspirin 75-125 mg	Mean (SD), 37.8 (10.7)	Oral contraceptives: 6 (LDA), 5 (LDA + W) IUD: not reported	Not reported
Matyas et al. [28], 2015	Prospective cohort study	United States	26 (71 person-days)	Self-reported OTC use, active ingredient aspirin	Median number of days consumed, 3 d	Mean (SD), 27.3 (8.2) (total cohort)	None	Based on participant recorded daily medication intake, in the total cohort, 54 women took >1 analgesic
Iudici et al. [29], 2016	Retrospective cohort study	Italy	143	Aspirin	Aspirin 100 mg daily	Mean (SD), 35.3 (13.4) (total cohort)	Not reported	Not reported
Devabhaktuni et al. [30], 2017	Prospective cohort study	India	11	Aspirin and/or clopidogrel	Not reported	21-30 y: 1 31-40 y: 1 41-50 y: 8 50-60 y: 1	None	Antiplatelet group: 6 Anticoagulant group: 22
Boonyawat et al. [31], 2021	Parallel-group, double-blind, randomized trial	20 countries	108	Aspirin	Aspirin 100 mg daily	Median (IQR), 39 (32-45)	Not specified	NSAID use at baseline: 0 (aspirin group)
Kawamatsu et al. [32], 2021	Retrospective cohort study	Japan	28	Not specified	Not reported	Mean (SD), 26 (6)	Not reported	Not reported
Rodpetch et al. [33], 2021	Cross-sectional study	Thailand	16	Aspirin or clopidogrel^c^	Aspirin 81 mg, clopidogrel 75 mg	Median (IQR), 40 (34-46) (total cohort)	Oral contraceptives (total cohort): 5 IUD: not reported DMPA: 1	Not reported
Matsushita et al. [34], 2022	Prospective cohort study	Japan	8	Not specified	Not reported	Median (range), 21.5 (16-39) (total cohort)	Not reported	Not reported

DMPA, depot medroxyprogesterone acetate; IUD, intrauterine device; LDA, low-dose aspirin; NSAID, nonsteroidal anti-inflammatory drug; OTC, over-the-counter; W, warfarin.

a Total number of women exposed to antiplatelet therapy.

b Age of women exposed to antiplatelet therapy, unless otherwise indicated.

c Only 1 included patient used clopidogrel.

**Table 2:** Characteristics of participants of included studies.

Author, year	Participant characteristics
Corson and Bolognese [22], 1978	Regularly cycling women with primary dysmenorrhea requiring analgesic use for pain relief for at least 1 y and for each of the 3 preceding cycles.
Hahn and Petruson [23], 1979	Regularly cycling women aged between 20 and 40 y, either using or not using a copper-containing IUD.
Krishna et al. [24], 1980	Regularly cycling women aged between 14 and26 y with primary dysmenorrhea for at least 3 mo.
Pedron et al. [25], 1987	Regularly cycling women who had a copper-containing IUD and who experienced increased menstrual blood loss.
Wun et al. [26], 2013	Adult patients with SCD (genotypes HbSS, HbSC, HbS-β0-thalassemia, and HbS-β+-thalassemia), aged 18-55 y, who did not have a diagnosis of acute VOC within 30 d of the study screening visit.
Cuadrado et al. [27], 2014	aPL-positive patients (ie, presence of aPLs on at least 2 occasions with an interval of 6 wk during the year prior to inclusion), SLE and/or pregnancy morbidity as defined by obstetric APS, aged between 18 and 65 y.
Matyas et al. [28], 2015	Regularly cycling women aged between 18 and 44 y.
Iudici et al. [29], 2016	Patients admitted to the rheumatology unit, satisfying the ACR criteria and/or SLICC criteria for SLE without a history of a cardiovascular event (angina, myocardial infarction, heart failure, TIA, stroke, or atherosclerotic peripheral ischemia).
Devabhaktuni et al. [30], 2017	Women treated with anticoagulants or antiplatelet agents who were referred to the gynecologist.
Boonyawat et al. [31], 2021	Women having menstrual cycles, aged ≥18 y, who had an objectively confirmed VTE and who had been treated for 6-12 mo with either a VKA or DOAC and had not interrupted therapy for >7 dbefore randomization, who did not require extended anticoagulant therapy at therapeutic dosage or antiplatelet therapy.
Kawamatsu et al. [32], 2021	Patients with Fontan circulation, aged ≥15 y.
Rodpetch et al. [33], 2021	Women who were treated with antiplatelet agents or oral anticoagulants, aged between 18 and 50 y, who had regular menstruation or at least once in the past 3 mo.
Matsushita et al. [34], 2022	Women who had been treated with Fontan surgery.

ACR, American College of Rheumatology; aPL, antiphospholipid antibodies; APS, antiphospholipid syndrome; DOAC, direct oral anticoagulant; IUD, intrauterine device; SCD, sickle cell disease; SLE, systemic lupus erythematosus; SLICC, Systemic Lupus International Collaborating Clinics; TIA, transient ischemic attack; VKA, vitamin K antagonist; VOC, vaso-occlusive crisis; VTE, venous thromboembolism.

Included studies used varying types of comparison groups: other types of anticoagulants (such as DOACs or VKAs) [[30], [31], [32], [33], [34]], aspirin combined with low-intensity VKA (target international normalized ratio, 1.5; range, 1.3-1.7) [27], placebo [26], nonantiplatelet drug users [28], or multiple comparison groups (placebo and paracetamol [23], placebo and ibuprofen [22], and placebo and flurbiprofen [24]). In 3 studies, in addition to other comparisons, menstrual blood loss was compared to menstrual cycles before receiving antiplatelet therapy in retrospect, as reported by participants [24,31,33]. One study determined the amount of menstrual blood loss during 1 pretreatment control cycle by the alkaline hematin method as a comparison [25]. Only 1 study had no comparison group or control cycle [29].

### 3.3 Risk of bias

**Figure 2:**
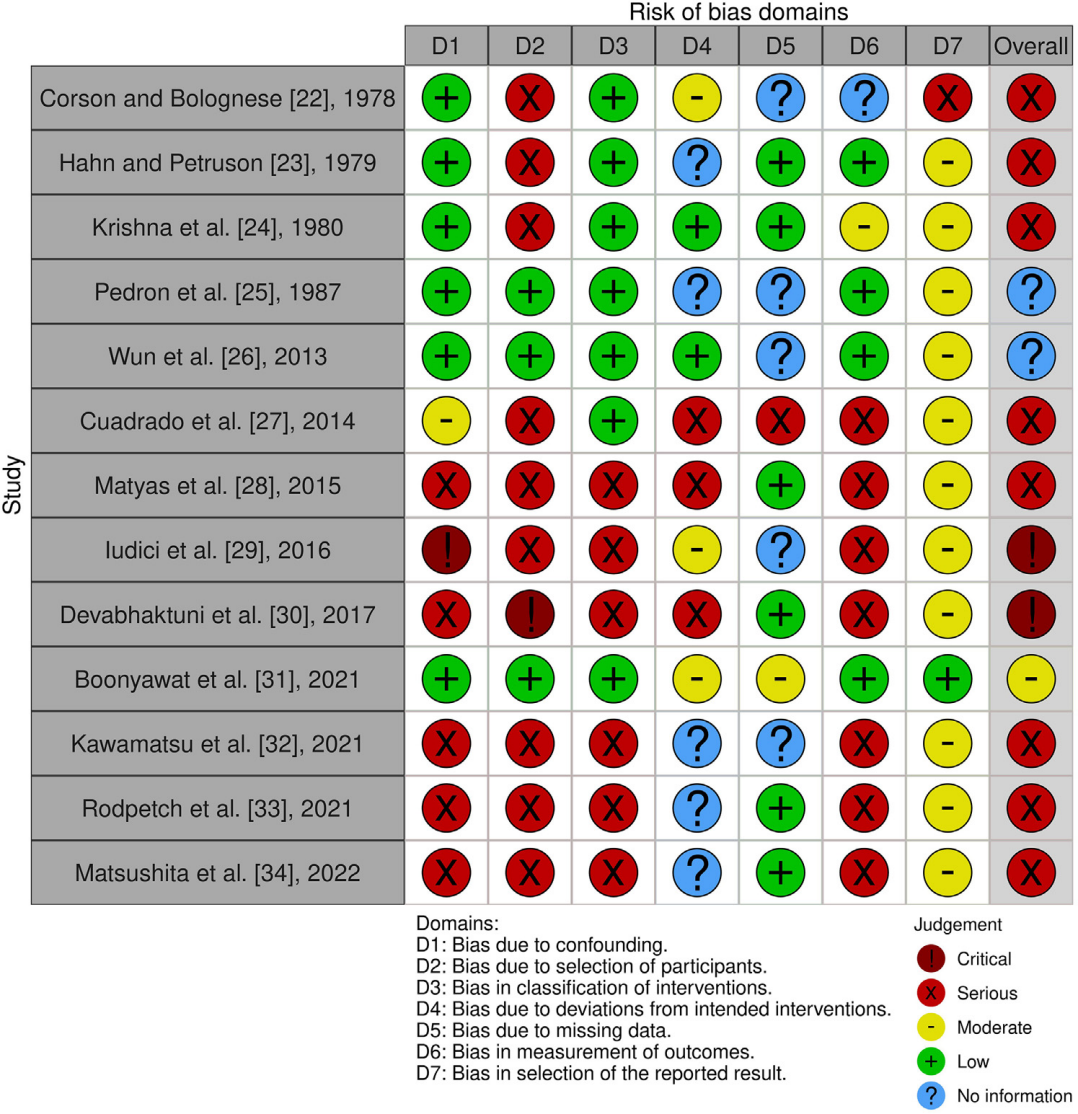
Risk of bias judgment. Both domain-level and overall risk of bias judgments for all included studies. This figure was created with the *robvis* tool [35].

Domain-level risk of bias as well as overall risk of bias judgments are displayed in Figure 2 [35].

The majority of included studies were assessed as being at serious risk of bias (8/13 studies), and 2 studies were assessed as being at critical risk of bias. Only 1 study was judged to be at moderate risk of bias. For 2 studies, the overall risk of bias judgment could not be assessed because of lack of information. According to the Risk of Bias in Non-randomised Studies - of Interventions tool, studies at critical risk of bias are “too problematic to provide any useful evidence and should not be included in any synthesis” [20]. Therefore, these studies [29,30] were excluded from the synthesis.

### 3.4 Results of individual studies

Multiple studies assessed different aspects of menstrual blood loss: changes in menstrual blood loss volume as measured in milliliters [23,25,28], duration of menstrual bleeding [28,31,33], intensity of menstrual flow as self-reported by women [22,24,28,31,33], and the prevalence or incidence of HMB [26,27,[32], [33], [34]]. Outcome measures related to menstrual blood loss, assessment methods, and results of all studies included in our synthesis are displayed in Supplementary Table S1.

Three studies assessed changes in menstrual blood loss volume [23,25,28] (Table 3). In total, these studies reported data on 284 cycles during which aspirin was used. Two of these studies used the alkaline hematin method to assess the menstrual blood loss volume [23,25]; the other used the menstrual pictogram [28]. One crossover study suggested no effect of aspirin on menstrual blood loss volume [23], whereas the other 2 studies suggested that aspirin may increase menstrual blood loss volume, primarily in women who did not experience HMB prior to their aspirin use [25,28]. The reported menstrual blood loss volume was larger among aspirin users than nonusers (mean ± SD, 53.7 ± 2.4 vs 45.4 ± 2.7 mL) [28].

**Table 3:** Study results on menstrual blood loss volume.

Author, year	Study design	Overall risk of bias^a^	Antiplatelet drug type and dose	Assessment method	Intervention		Comparison			
Hahn and Petruson [23], 1979	Nonrandomized double-blind crossover study	Serious	Aspirin 500 mg (starting on the first day of menstruation, 1 tablet 3 times a day, continued during the bleeding period)	Alkaline hematin method with the use of collected sanitary towels and tampons	Aspirin^b^		Paracetamol^b^		Placebo^b^	
					No IUD (*N* = 23): 57.4 mL (SD, ±47)	IUD (*N* = 10): 49.2 mL (SD, ±30)	No IUD (*N* = 23): 55.6 mL (SD, ±44)	IUD (*N* = 10): 49.5 mL (SD, ±29)	No IUD (*N* = 23): 49.3 mL (SD, ±45)	IUD (*N* = 10): 50.7 mL (SD, ±34)
Pedron et al. [24], 1987	Prospective intervention study	No information	Aspirin 500 mg (2 tablets every 8 h from the onset of menstrual bleeding for 5 d)	Alkaline hematin method with the use of collected sanitary towels and tampons	Aspirin^c^		Preaspirin control cycle			
					<60 mL (*N* = 24): change in menstrual blood loss ranged from +51.4% up to +85%		<60 mL: 24 women			
					60-80 mL (*N* = 16): change in menstrual blood loss ranged from −7.5% up to +22.4% (*N* = 16)		60-80 mL: 16 women			
					≥80 mL (*N* = 13): change in menstrual blood loss ranged from −17.6% upto +5.9%		≥80 mL: 13 women			
Matyas et al. [28], 2015	Prospective cohort study	Serious	OTC aspirin, self-reported median 3 d consumed	Menstrual pictogram from Wyatt et al. [36] filled in by participants	Cycles with aspirin use (OTC, self-reported): mean, 53.7 mL (SD, ±2.4) (*N* = 39 cycles)		Cycles without aspirin use: mean, 45.4 mL (SD, ±2.7) (*N* = 470 cycles)			

IUD, intrauterine device; OTC, over-the-counter.

a Overall risk of bias judgments based on the Risk of Bias in Non-randomised Studies - of Interventions assessment tool.

b Mean menstrual blood loss volume per menstrual cycle.

c Percentage change in menstrual blood loss volume from the preaspirin control cycle over 4 consecutive menstrual cycles.

Three studies assessed the duration of menstrual bleeding [28,31,33] (Table 4). Of these, 1 study reported a longer duration of menstruation among aspirin users (mean ± SD, 7.9 ± 2.4 days) than among nonusers (mean ± SD, 6.9 ± 2.2 days) [28]. In the other 2 studies, 9% to 12.5% of women reported an increased duration of menstruation compared to their menstruation prior to antiplatelet therapy [31,33]. These percentages were comparable to or lower than the anticoagulant comparison groups. Among women randomized to rivaroxaban 20 mg, 12% to 18% reported an increased menstrual flow duration during a 1-year follow-up period. This was 6% to 12% among women randomized to rivaroxaban 10 mg. Among VKA users, 41% of women reported an increased duration of menstruation compared to prior anticoagulant therapy.

**Table 4:** Study results on duration of menstrual bleeding.

Author, year	Study design	Overall risk of bias^a^	Antiplatelet drug type and dose	Intervention	Comparison	
Matyas et al. [28], 2015	Prospective cohort study	Serious	OTC aspirin, self-reported median 3 d consumed	Cycles with aspirin use (OTC, self-reported): mean, 7.9 d (SD, ±2.4 d) (*N* = 39 cycles)	Cycles without aspirin use: mean, 6.9 d (SD, ±2.2 d) (*N* = 470 cycles)	
Boonyawat et al. [31], 2021	Parallel-group, double-blind,randomized trial	Moderate	Aspirin 100 mg daily	Aspirin (*N* = 108)^b^	Rivaroxaban 20 mg (*N* = 134)^b^	Rivaroxaban 10 mg (*N* = 120)^b^
				9%-12% ↑ duration	12%-18% ↑ duration	6%-12% ↑ duration
				Increased menstrual flow duration: Rivaroxaban 20 mg vs aspirin: OR, 1.36; 95% CI, 0.62-2.96 Rivaroxaban 10 mg vs aspirin: OR, 0.77; 95% CI, 0.33-1.81		
Rodpetch et al. [33], 2021	Cross-sectional study	Serious	Aspirin 81 mg, clopidogrel 75 mg	Antiplatelet (N = 16)	VKA (N = 29)	DOAC (N = 4)
				4.6 d (SD, ±1.9 d) before and after therapy^c^	4.5 (SD, ±2.0 d) before vs 5.2 (SD, ±2.4 d) after therapy c	2.8 (SD, ±1.7 d) before vs 4 (SD, ±1.2 d) after therapy c
				12.5% ↑ duration	41.1% ↑ duration	50% ↑ duration

DOAC, direct oral anticoagulant; OR, odds ratio; OTC, over-the-counter; VKA, vitamin K antagonist.

a Overall risk of bias judgments based on the Risk of Bias in Non-randomised Studies - of Interventions assessment tool.

b Menstrual flow duration versus prior any anticoagulant therapy, over 5 follow-up visits during the 1-year follow-up period

c Mean (±SD) duration of menstrual bleeding before and after antiplatelet or anticoagulant therapy.

Five studies assessed the intensity of menstrual flow [22,24,28,31,33] (Table 5). In all studies, menstrual flow intensity was self-reported by participants. In 3 studies, participants were asked to compare their menstrual flow intensity to their menstruation before taking antithrombotic or study treatment [24,31,33]. In 2 of these studies, 13% to 38% of women reported an increased intensity of menstrual flow during antiplatelet therapy [31,33]. In comparison, among women randomized to either rivaroxaban 20 mg or rivaroxaban 10 mg, 19% to 24% and 14% to 21%, respectively, reported increased menstrual flow intensity during a 1-year follow-up period. Among VKA users, 66% reported increased menstrual flow intensity. One study reported a higher proportion of women who experienced their menstrual flow as heavy among aspirin users (42%) compared with nonusers (32%) [28]. In contrast, 2 crossover studies noted no differences in menstrual flow between aspirin and ibuprofen and placebo treatment cycles [22,24].

**Table 5:** Study results on intensity of menstrual flow.

Author, year	Study design	Overall risk of bias^a^	Antiplatelet drug type and dose	Assessmentmethod	Intervention	Comparison		
Corson and Bolognese [22], 1978	Randomized double-blind crossover study	Serious	Aspirin 325 mg (2 tablets every 4 h as necessary for pain relief)	Self-reported by participants	Aspirin (*N* = 33)	Ibuprofen (*N* = 33)		Placebo (*N* = 33)
					No differences in menstrual flow between treatment groups			
Krishna et al. [24], 1980	Nonrandomized double-blind crossover study	Serious	Aspirin 300 mg (2 tablets at onset of dysmenorrhea, continued at 8 h intervals until symptoms disappeared)	Self-recorded menstrual blood loss: whether more, less, or the same as compared to pretrial menstrual blood loss	Aspirin	Placebo		
					5/39 women ↑ menstrual blood loss vs pretrial blood loss	6/39 women ↑ menstrual blood loss vs pretrial blood loss		
Matyas et al. [28], 2015	Prospective cohort study	Serious	OTC aspirin, self-reported median 3 d consumed	Menstrual pictogram from Wyatt et al. [36] filled in by participants	Cycles with aspirin use (OTC, self-reported): 42% heavy menstrual flow (*N* = 39 cycles)	Cycles without aspirin use: 32% heavy menstrual flow (*N* = 470 cycles)		
Boonyawat et al. [31], 2021	Parallel-group, double-blind, randomized trial	Moderate	Aspirin 100 mg daily	Self-reported by participants, comparing menstrual flow intensity of their last menstruation with their menstruation before the start of any anticoagulant therapy (less than usual, as usual, or more than usual)	Aspirin (*N* = 108)^b^	Rivaroxaban 20 mg (*N* = 134) b	Rivaroxaban 10 mg (*N* = 120) b	
					13%-20% ↑ flow intensity	19%-24% ↑ flow intensity	14%-21% ↑ flow intensity	
					*Increased menstrual flow intensity:* Rivaroxaban 20 mg vs aspirin: OR, 1.41; 95% CI, 0.67-2.99 Rivaroxaban 10 mg vs aspirin: OR, 1.07; 95% CI, 0.49-2.34			
Rodpetch et al. [33], 2021	Cross-sectional study	Serious	Aspirin 81 mg, clopidogrel 75 mg	Self-reported by participants change in intensity of menstrual bleeding before and after receiving oral antithrombotics	Antiplatelet (*N* = 16)^c^	VKA (*N* = 29)^c^	DOAC (*N* = 4)^c^	
					37.5% ↑ flow intensity	65.5% ↑ flow intensity	75% ↑ flow intensity	

DOAC, direct oral anticoagulant; OR, odds ratio; OTC, over-the-counter; VKA, vitamin K antagonist.

a Overall risk of bias judgments based on the Risk of Bias in Non-randomised Studies - of Interventions assessment tool.

b Menstrual flow intensity versus prior any anticoagulant therapy, over 5 follow-up visits during the 1-year follow-up period.

c Self-reported increased menstrual flow intensity versus prior antiplatelet or anticoagulant therapy.

Five studies reported a prevalence or incidence estimate of HMB [26,27,[32], [33], [34]] (Table 6). The number of women who received antiplatelet therapy varied between 8 and 80 across studies. In total, these studies included 153 women exposed to antiplatelet therapy. Applied definitions and assessment methods differed across studies, but most often, authors reported on the occurrence of menorrhagia, which was not further specified. The 60-day cumulative incidence of menorrhagia in patients with sickle cell disease randomized to prasugrel was 9.5% vs 0% in patients randomized to placebo [26]. During a median follow-up of 3 years, no menorrhagia was reported by patients with antiphospholipid antibodies randomized to aspirin, whereas 12.5% of patients randomized to aspirin combined with low-intensity VKA therapy reported menorrhagia [27].

**Table 6:** Study results on the prevalence or incidence of heavy menstrual bleeding.

Author, year	Study design	Overall risk of bias^a^	Antiplatelet drug type and dose	Menorrhagia: definitionand assessment method	Intervention	Comparison			
Wun et al. [26], 2013	Randomized double-blind controlled phase 2 trial	No information	Prasugrel 5 mg daily	Menorrhagia not further specified Either events requiringmedical attention or recorded in retrospect during study visits by interviewing patients	Prasugrel	Placebo			
					60 d incidence of menorrhagia in patients with SCD: 2/21 (9.5%)	60 d incidence of menorrhagia in patients with SCD: 0/9			
Cuadrado et al. [27], 2014	Randomized open-label controlled trial	Serious	Aspirin 75-125 mg	Menorrhagia not further specified Assessed by questionnaire at study visits or from eneral physician/hospital reports	Aspirin	Aspirin + low-intensity VKA^b^			
					aPL-positive patients, median FU 3 y: 0/80 reported menorrhagia	aPL-positive patients, median FU 3 y: 10/80 (12.5%) reported menorrhagia			
Kawamatsu et al. [32], 2021	Retrospective cohort study	Serious	Not reported	Menorrhagia not further specified Based on data collected from electronic medical records	Antiplatelet	DOAC	VKA		Antiplatelet + anticoagulant
					Patients with Fontan circulation: 2/28 (7.1%) menorrhagia	Patients with Fontan circulation: 1/16 (6.3%) menorrhagia	Patients with Fontan circulation: 4/16 (25%) menorrhagia		Patients with Fontan circulation: 5/10 menorrhagia
Rodpetch et al. [33], 2021	Cross-sectional study	Serious	Aspirin 81 mg, clopidogrel 75 mg	Using the subjective definition of HMB as the gold standard, an MBQ score of ≥21.5 wasused to define HMB (sensitivity 82.9%; specificity 83.1%)	Antiplatelet (*N* = 16)	VKA (*N* = 29)		DOAC (*N* = 4)	
					25.0% (95% CI, 7.0-52.0)	27.6% (95% CI, 12.7-47.0)		25.0% (95% CI, 0.6-80.0)	
Matsushita et al. [34], 2022	Prospective cohort study	Serious	Not reported	HMB is defined as the usual rate of changing pads during full flow higher than 8 times daily Based on a structured questionnaire	Antiplatelet	Anticoagulant		Combination	
					Women with Fontan circulation: 3/8 HMB	Women with Fontan circulation: 2/8 HMB		Women with Fontan circulation: 2/2 HMB	

aPL, antiphospholipid antibodies; DOAC, direct oral anticoagulant; FU, follow-up; HMB, heavy menstrual bleeding; MBQ, menstrual bleeding questionnaire; SCD, sickle cell disease; VKA, vitamin K antagonist.

a Overall risk of bias judgments based on the Risk of Bias in Non-randomised Studies - of Interventions assessment tool.

b Target international normalized ratio, 1.5 (range, 1.3-1.7).

### 3.5 Chronic antiplatelet therapy

Six studies reported on the chronic use of antiplatelet drugs for primary or secondary cardiovascular disease prevention [26,27,[31], [32], [33], [34]] (Supplementary Table S2). Participants were treated with prasugrel (5 mg daily) [26], aspirin (dosage range, 75-125 mg daily) [27,31], aspirin or clopidogrel (aspirin 81 mg, clopidogrel 75 mg daily) [33], and unspecified antiplatelet drugs [32,34]. Two studies reported that chronic use of aspirin may, in a minority of women, increase menstrual flow duration and/or self-perceived menstrual flow intensity [31,33]. The other studies only described the occurrence of HMB or menorrhagia in their study population. These estimates ranged from 0 of 80 among antiphospholipid antibody–positive patients during a median follow-up of 3 years [27], 2 of 21 among patients with sickle cell disease within a follow-up period of 60 days [26], 2 of 28 in patients with Fontan circulation [32], 3 of 8 in patients with Fontan circulation [34], and 4 of 16 among women who received antiplatelet therapy for various indications [33].

## 4 Discussion

In this systematic review, we identified 13 studies, at high risk of bias, that examined the effects of antiplatelet therapy on menstrual blood loss. Most studies only partially or indirectly addressed our review question. Some studies suggested that antiplatelet therapy increased menstrual blood loss, whereas others showed no effect. However, studies judged as being at the lowest risk of bias suggested that, in some women, the use of aspirin does increase menstrual blood loss in terms of volume, duration, or experienced flow intensity. This effect may be limited to women who did not suffer from HMB before their aspirin use. P2Y12 inhibitors have not been sufficiently studied to conclude on their effects on menstrual blood loss.

The effects of high dosages of aspirin as intended therapy for dysmenorrhea, restricted to the period of menstruation only, should be distinguished from the effects of chronic use of low-dose aspirin for cardiovascular indications. It has been hypothesized that aspirin could possibly decrease menstrual blood loss by its inhibiting effect on prostaglandin synthesis [23]. Therefore, in 4 studies the use of aspirin was restricted to the period of menstruation in order to examine its possible decreasing effect on menstrual blood loss and/or its efficacy as therapy for dysmenorrhea. These differences in the use of aspirin may also have affected the obtained results regarding the effects on menstrual blood loss and may explain why inconsistent effects were observed across studies.

Likewise, different assessment methods for menstrual blood loss may have influenced the obtained results. Several studies have reported discrepancies between subjective and objective measures of menstrual blood loss [10,37]. Some of the included studies used the alkaline hematin technique, whereas other estimates were based on self-reported measures. These self-reported measures are more prone to bias, for example recall bias. This is especially the case when participants are asked to compare their menstrual bleeding with that before receiving antithrombotic therapy, which was the case in 3 of our included studies. Nevertheless, self-reported measures of menstrual blood loss remain important to capture the effects on quality of life, as also highlighted by the International Federation of Gynecology and Obstetrics' definition of HMB [6,7].

The effects of oral anticoagulants on menstrual blood loss are better characterized and seem more pronounced than the effects of antiplatelet therapy. Several studies have reported that the use of oral anticoagulants, such as DOACs or VKAs, is associated with HMB, higher PBAC scores, and lower menstrual bleeding–specific quality of life [38]. A prospective cohort study among women with an active menstrual cycle who had a (recurrent) VTE and were treated with anticoagulants (87% DOACs and 12% VKAs) reported that during a 6-month follow-up period, 66% (95% CI, 57%-75%; 65/98 women) of women met at least once 1 of the criteria of the author’s definition of AUB (a PBAC score of >100 or self-reported increased menstrual volume) [4]. The incidence of new-onset AUB was estimated at 60% (36 of 60; 95% CI, 47%-71%) among women who were considered not to have AUB prior to anticoagulant treatment, which was determined in retrospect with the PBAC referring to the woman’s last menstrual period before VTE [4]. Some studies even suggest that HMB occurs more frequently in women treated with factor Xa inhibitors compared with VKAs [16,[39], [40], [41]]. In contrast, the thrombin inhibitor dabigatran has been associated with lower risk of HMB than VKAs [42].

Effects of antiplatelet therapy on other bleeding complications have also been described. A recent meta-analysis showed that prophylactic use of low-dose aspirin for the prevention of preeclampsia in pregnant women is associated with a 24% increased risk of postpartum hemorrhage [43]. Moreover, aspirin use for primary prevention of cardiovascular events has been associated with increased rate of major bleeding, including intracranial hemorrhage and major gastrointestinal bleeding, compared with no aspirin (hazard ratio, 1.43; 95% credible interval, 1.30-1.56) [44]. The study population consisted of 52.8% women; however, the mean age at study entry ranged between 53 and 74 years. These findings may, therefore, not be generalizable to the population of reproductive-aged women. Both clopidogrel as well as aspirin monotherapy for secondary prevention of cardiovascular disease have been associated with similar odds of major bleeding [45]. However, these studies have been conducted in cohorts that primarily consisted of males (only 26% women) with a higher average age (62.7 ±11 years).

The bleeding risk associated with aspirin therapy has also been compared to anticoagulant therapy. For example, the Rivaroxaban or Aspirin for Extended Treatment of Venous Thromboembolism (EINSTEIN-CHOICE) trial reported similar risks of major and clinically relevant nonmajor bleeding associated with aspirin compared with rivaroxaban, also in the subgroup of women [46]. Likewise, the rate of major bleeding was comparable among patients with atrial fibrillation taking apixaban compared to aspirin in the Apixaban Versus Acetylsalicylic Acid to Prevent Stroke in Atrial Fibrillation Patients Who Have Failed or Are Unsuitable for Vitamin K Antagonist Treatment (AVERROES) trial, also in the subgroup analysis restricted to women [47]. However, the average age of participants in the AVERROES trial was 70 years. Nevertheless, aspirin has been associated with lower risk of major bleeding compared with VKAs in patients with atrial fibrillation, heart failure, or stroke [48].

The evidence included in this review has some limitations. First, the majority of included studies had a limited sample size. The number of women exposed to antiplatelet drugs ranged from 11 to 143, leading to imprecise estimates. Second, only 3 studies included women who used nonaspirin antiplatelet drugs. The effects of P2Y12 inhibitors, such as clopidogrel, prasugrel, and ticagrelor, on menstrual blood loss could differ from the effects of aspirin and could, therefore, not be reliably assessed. Third, there were major concerns about the risk of bias in the included studies. The majority of included studies were assessed as being at serious or higher risk of bias, especially risk of selection bias and confounding bias, in particular confounding by age. For example, the underlying pathology of HMB may vary with age as the risk of uterine structural abnormalities increases with age [49]. Moreover, some women may have had an undiagnosed mild bleeding diathesis, which could have contributed to the effects of antiplatelet drugs on menstrual blood loss. Furthermore, some studies failed to control for important cointerventions, such as the use of oral contraceptives or IUD and concomitant use of nonsteroidal anti-inflammatory drugs. In addition, the definition of menorrhagia was often not reported and, if reported, inconsistent definitions were used across studies. Possible underestimation of the occurrence of menorrhagia cannot be ruled out because of the lack of systematic assessment and noncomparability of outcome assessment methods across intervention groups. Finally, limitations of the review process include restriction to English studies only and the impossibility of performing a formal meta-analysis because of heterogeneity in outcome reporting of the included studies and insufficient data.

Findings from this review highlight that there are limited data available on the effects of antiplatelet therapy on menstrual blood loss. Future studies on the effects of antiplatelet therapy on menstrual blood loss should not be restricted to aspirin but should also include nonaspirin antiplatelet drugs, the most important of which are P2Y12 inhibitors. In addition, outcomes should not be limited to objective measures of menstrual blood loss, but also the perspective and experiences of the women themselves and possible effects of increased menstrual blood loss on menstrual bleeding–specific quality of life should be explored. Currently, the PBAC is the most often used tool to measure menstrual blood loss in clinical studies [12], but validated instruments to capture the menstrual bleeding–specific quality of life are also available [50]. Ideally, further standardization of assessment methods for menstrual blood loss and effects on quality of life will be performed. Hence, if HMB also represents an issue for premenopausal women treated with antiplatelet drugs, future studies can contribute to increasing awareness among healthcare professionals and patients and eventually improving care for these women by mitigating the consequences of HMB.

## 5 Conclusion

To conclude, this is the first systematic review on the effects of antiplatelet therapy on menstrual blood loss. Aspirin may increase menstrual blood loss, at least in a minority of women. There is lack of data regarding the effects of P2Y12 inhibitors on menstrual blood loss. Therefore, additional studies are needed to assess the effects of different types of antiplatelet drugs on menstrual blood loss and to examine whether these possible adverse effects represent an important issue for female antiplatelet drug users.
