# Supplementary material for: Effects of antiplatelet therapy on menstrual blood loss in reproductive-aged women: a systematic review
Source: Res Pract Thromb Haemost. 2023 Dec 9;8(1):102295. doi: 10.1016/j.rpth.2023.102295 (PMC10805676; doi:10.1016/j.rpth.2023.102295)
Supplement: Supplementary Tables [file mmc1.pdf]

Table of contents

Search strategy 2

Item 16b of PRISMA 2020 checklist 5

Supplementary table S1. Study results on chronic antiplatelet therapy 6

Supplementary table S2. Outcomes and assessment methods of menstrual blood loss 6

References 13

Search strategy

Database searched	Platform	Years of coverage	Records	Records after duplicates removed
Medline ALL	Ovid	1946 - Present	74	74
Embase	Embase.com	1971 - Present	513	445
Web of Science Core Collection*	Web of Knowledge	1975 - Present	39	9
Cochrane Central Register of Controlled Trials**	Wiley	1992 - Present	16	7
Additional Search Engines: Google Scholar***			100	72
**Total**			**742**	**607**

*Science Citation Index Expanded (1975-present) ; Social Sciences Citation Index (1975-present) ; Arts & Humanities Citation Index (1975-present) ; Conference Proceedings Citation Index- Science (1990-present) ; Conference Proceedings Citation Index- Social Science & Humanities (1990-present) ; Emerging Sources Citation Index (2005-present)

** Manually deleted abstracts from trial registries

***Google Scholar was searched via "Publish or Perish" to download the results in EndNote.

No other database limits were used than those specified in the search strategies

Medline 74

(Menorrhagia/ OR ((Menstruation / OR Menstruation Disturbances /) AND Hemorrhage /) OR (((menstrua*) ADJ3 (heavy OR excessive* OR blood-loss OR bleeding)) OR hypermenor* OR menor* OR menometror* OR metror*).ab,ti.) AND (exp Platelet Aggregation Inhibitors/ OR Dual Anti-Platelet Therapy / OR (antithrombocyt* OR anti-thrombocyt* OR antiplatelet* OR anti-platelet* OR ((platelet* OR thrombocyt*) ADJ3 aggregat* ADJ3 inhibit*) OR ((Fibrinogen* OR thrombin*) ADJ3 receptor* ADJ3 antagonist*) OR acetylsalicylic* OR ajoen* OR aloxiprin* OR alprostadil-alfadex* OR anagrelid* OR ancrod* OR applaggin* OR aprosulat* OR aspalaton* OR ataprost* OR atopaxar* OR beraprost* OR buflomedil-pyridoxal-phosphat* OR cangrelor* OR caplacizumab* OR cicaprost* OR cilostazol* OR ciprosten* OR clopidogrel* OR cryptolepin* OR d-003* OR dazoxiben* OR dehydrocilostazol* OR dermatan* OR dermatan-sulfat* OR dextran-40* OR dipyridamol* OR dipyridamole-tl-201* OR elinogrel* OR enfenamic-acid* OR esuberaprost* OR glenzocimab* OR heparan-sulfat* OR ifetroban* OR iloprost* OR imolamin* OR indobufen* OR isbogrel* OR itazigrel* OR linotroban* OR lixazinon* OR mipitroban* OR nafazatrom* OR naxaprosten* OR octimibat* OR oxagrelat* OR pamicogrel* OR pentosan-polysulfat* OR pentoxifyllin* OR picotamid* OR piracetam* OR plafibrid* OR prasugrel* OR prostacyclin* OR rafigrelid* OR regrelor* OR samixogrel* OR sarpogrelat* OR satigrel* OR sc-38249* OR selatogrel* OR sulfinpyrazon* OR taprosten* OR temanogrel* OR terbogrel* OR terutroban* OR ticagrelor* OR ticlopidin* OR treprostinil* OR triflusal* OR trombodipin* OR (arginylglycylaspartyl ADJ3 amid*) OR abciximab* OR albolabrin* OR arginylglycylaspartylserin* OR bitistatin* OR carafiban* OR contortrostatin* OR disintegrin* OR echistatin* OR elarofiban* OR eptifibatid* OR fradafiban* OR gantofiban* OR glycylarginylglycylaspartylserin* OR kistrin* OR lamifiban* OR lefradafiban* OR lotrafiban* OR orbofiban* OR roxifiban* OR sibrafiban* OR tadocizumab* OR tirofiban* OR triflavin* OR

trigramin* OR xemilofiban* OR zalunfiban* OR vorapaxar*).ab,ti.) NOT (exp animals/ NOT humans/) AND english.la.

embase.com 513

('menorrhagia and metrorrhagia'/de OR menorrhagia/de OR ((menstruation/de OR 'menstruation disorder'/de) AND bleeding/de) OR (((menstrua*) NEAR/3 (heavy OR excessive* OR blood-loss OR bleeding)) OR hypermenor* OR menor* OR menometror* OR metror*):ab,ti) AND ('antithrombocytic agent'/exp OR 'dual antiplatelet therapy'/de OR (antithrombocyt* OR anti-thrombocyt* OR antiplatelet* OR anti-platelet* OR ((platelet* OR thrombocyt*) NEAR/3 aggregat* NEAR/3 inhibit*) OR ((Fibrinogen* OR thrombin*) NEAR/3 receptor* NEAR/3 antagonist*) OR acetylsalicylic* OR ajoen* OR aloxiprin* OR alprostadil-alfadex* OR anagrelid* OR ancrod* OR applaggin* OR aprosulat* OR aspalaton* OR ataprost* OR atopaxar* OR beraprost* OR buflomedil-pyridoxal-phosphat* OR cangrelor* OR caplacizumab* OR cicaprost* OR cilostazol* OR ciprosten* OR clopidogrel* OR cryptolepin* OR d-003* OR dazoxiben* OR dehydrocilostazol* OR dermatan* OR dermatan-sulfat* OR dextran-40* OR dipyridamol* OR dipyridamole-tl-201* OR elinogrel* OR enfenamic-acid* OR esuberaprost* OR glenzocimab* OR heparan-sulfat* OR ifetroban* OR iloprost* OR imolamin* OR indobufen* OR isbogrel* OR itazigrel* OR linotroban* OR lixazinon* OR mipitroban* OR nafazatrom* OR naxaprosten* OR octimibat* OR oxagrelat* OR pamicogrel* OR pentosan-polysulfat* OR pentoxifyllin* OR picotamid* OR piracetam* OR plafibrid* OR prasugrel* OR prostacyclin* OR rafigrelid* OR regrelor* OR samixogrel* OR sarpogrelat* OR satigrel* OR sc-38249* OR selatogrel* OR sulfinpyrazon* OR taprosten* OR temanogrel* OR terbogrel* OR terutroban* OR ticagrelor* OR ticlopidin* OR treprostinil* OR triflusal* OR trombodipin* OR (arginylglycylaspartyl NEAR/3 amid*) OR abciximab* OR albolabrin* OR arginylglycylaspartylserin* OR bitistatin* OR carafiban* OR contortrostatin* OR disintegrin* OR echistatin* OR elarofiban* OR eptifibatid* OR fradafiban* OR gantofiban* OR glycylarginylglycylaspartylserin* OR kistrin* OR lamifiban* OR lefradafiban* OR lotrafiban* OR orbofiban* OR roxifiban* OR sibrafiban* OR tadocizumab* OR tirofiban* OR triflavin* OR trigramin* OR xemilofiban* OR zalunfiban* OR vorapaxar*):Ab,ti) NOT ([animals]/lim NOT [humans]/lim) AND [english]/lim

Web of science 39

TS=(((((menstrua*) NEAR/2 (heavy OR excessive* OR blood-loss OR bleeding)) OR hypermenor* OR menor* OR menometror* OR metror*)) AND ((antithrombocyt* OR anti-thrombocyt* OR antiplatelet* OR anti-platelet* OR ((platelet* OR thrombocyt*) NEAR/2 aggregat* NEAR/2 inhibit*) OR ((Fibrinogen* OR thrombin*) NEAR/2 receptor* NEAR/2 antagonist*) OR acetylsalicylic* OR ajoen* OR aloxiprin* OR alprostadil-alfadex* OR anagrelid* OR ancrod* OR applaggin* OR aprosulat* OR aspalaton* OR ataprost* OR atopaxar* OR beraprost* OR buflomedil-pyridoxal-phosphat* OR cangrelor* OR caplacizumab* OR cicaprost* OR cilostazol* OR ciprosten* OR clopidogrel* OR cryptolepin* OR d-003* OR dazoxiben* OR dehydrocilostazol* OR dermatan* OR dermatan-sulfat* OR dextran-40* OR dipyridamol* OR dipyridamole-tl-201* OR elinogrel* OR enfenamic-acid* OR esuberaprost* OR glenzocimab* OR heparan-sulfat* OR ifetroban* OR iloprost* OR imolamin* OR indobufen* OR isbogrel* OR itazigrel* OR linotroban* OR lixazinon* OR mipitroban* OR nafazatrom* OR naxaprosten* OR octimibat* OR oxagrelat* OR pamicogrel* OR pentosan-polysulfat* OR pentoxifyllin* OR picotamid* OR piracetam* OR plafibrid* OR prasugrel* OR prostacyclin* OR rafigrelid* OR regrelor* OR samixogrel* OR sarpogrelat* OR satigrel* OR sc-38249* OR selatogrel* OR sulfinpyrazon* OR taprosten* OR

temanogrel* OR terbogrel* OR terutroban* OR ticagrelor* OR ticlopidin* OR treprostinil* OR triflusal* OR trombodipin* OR (arginylglycylaspartyl NEAR/2 amid*) OR abciximab* OR albolabrin* OR arginylglycylaspartylserin* OR bitistatin* OR carafiban* OR contortrostatin* OR disintegrin* OR echistatin* OR elarofiban* OR eptifibatid* OR fradafiban* OR gantofiban* OR glycylarginylglycylaspartylserin* OR kistrin* OR lamifiban* OR lefradafiban* OR lotrafiban* OR orbofiban* OR roxifiban* OR sibrafiban* OR tadocizumab* OR tirofiban* OR triflavin* OR trigramin* OR xemilofiban* OR zalunfiban* OR vorapaxar*))) NOT DT=(Meeting Abstract OR Meeting Summary) AND LA=(english)

Cochrane 16

((((menstrua*) NEAR/3 (heavy OR excessive* OR blood NEXT loss OR bleeding)) OR hypermenor* OR menor* OR menometror* OR metror*):ab,ti) AND ((antithrombocyt* OR anti NEXT thrombocyt* OR antiplatelet* OR anti NEXT platelet* OR ((platelet* OR thrombocyt*) NEAR/3 aggregat* NEAR/3 inhibit*) OR ((Fibrinogen* OR thrombin*) NEAR/3 receptor* NEAR/3 antagonist*) OR acetylsalicylic* OR ajoen* OR aloxiprin* OR alprostadil NEXT alfadex* OR anagrelid* OR ancrod* OR applaggin* OR aprosulat* OR aspalaton* OR ataprost* OR atopaxar* OR beraprost* OR buflomedil NEXT pyridoxal NEXT phosphat* OR cangrelor* OR caplacizumab* OR cicaprost* OR cilostazol* OR ciprosten* OR clopidogrel* OR cryptolepin* OR d NEXT 003* OR dazoxiben* OR dehydrocilostazol* OR dermatan* OR dermatan NEXT sulfat* OR dextran NEXT 40* OR dipyridamol* OR dipyridamole NEXT tl NEXT 201* OR elinogrel* OR enfenamic NEXT acid* OR esuberaprost* OR glenzocimab* OR heparan NEXT sulfat* OR ifetroban* OR iloprost* OR imolamin* OR indobufen* OR isbogrel* OR itazigrel* OR linotroban* OR lixazinon* OR mipitroban* OR nafazatrom* OR naxaprosten* OR octimibat* OR oxagrelat* OR pamicogrel* OR pentosan NEXT polysulfat* OR pentoxifyllin* OR picotamid* OR piracetam* OR plafibrid* OR prasugrel* OR prostacyclin* OR rafigrelid* OR regrelor* OR samixogrel* OR sarpogrelat* OR satigrel* OR sc NEXT 38249* OR selatogrel* OR sulfinpyrazon* OR taprosten* OR temanogrel* OR terbogrel* OR terutroban* OR ticagrelor* OR ticlopidin* OR treprostinil* OR triflusal* OR trombodipin* OR (arginylglycylaspartyl NEAR/3 amid*) OR abciximab* OR albolabrin* OR arginylglycylaspartylserin* OR bitistatin* OR carafiban* OR contortrostatin* OR disintegrin* OR echistatin* OR elarofiban* OR eptifibatid* OR fradafiban* OR gantofiban* OR glycylarginylglycylaspartylserin* OR kistrin* OR lamifiban* OR lefradafiban* OR lotrafiban* OR orbofiban* OR roxifiban* OR sibrafiban* OR tadocizumab* OR tirofiban* OR triflavin* OR trigramin* OR xemilofiban* OR zalunfiban* OR vorapaxar*):Ab,ti)

Google scholar 100

"heavy|excessive menstrual|mentruation"|hypermenorrhagia|menorrhagia antithrombocytic|"anti-thrombocytic|platelet"|antiplatelet|"platelet|thrombocyte aggregation inhibitor|inhibitors"

Item 16b of PRISMA 2020 checklist:

Cite studies that might appear to meet the inclusion criteria, but which were excluded, and explain why they were excluded

Studies were excluded from our review because menstrual blood loss was not assessed [1-13], participants did not receive antiplatelet therapy [14-16], foreign language [17], use of other anticoagulants [18-23], population at risk (i.e. the proportion of reproductive-aged female study participants) could not be defined [24], results were not stratified by anticoagulant type [25-27], study design (non-systematic review [28-37], case report [38, 39] or case series with only 1 woman exposed to antiplatelet therapy [40]) or systematic reviews of which the studies were already included [41, 42].

Supplementary table S1. Outcomes and assessment methods of menstrual blood loss

Author (year)	Outcome measure related to MBL	Assessment method & definition	Intervention		Comparison			
**Corson (1978)**	Intensity of menstrual flow	Self-reported by participants	*Aspirin (N=33)*		*Ibuprofen (N=33)*		*Placebo (N=33)*	
			No differences in menstrual flow between treatment groups					
**Hahn (1979)**	Menstrual blood loss volume	Alkaline hematin method	*Aspirin**		*Paracetamol**		*Placebo**	
			No IUD (N=23): 57.4 ml (±47 SD)	IUD (N=10): 49.2 ml (±30 SD)	No IUD (N=23): 55.6 ml (±44 SD)	IUD (N=10): 49.5 ml (±29 SD)	No IUD (N=23): 49.3 ml (±45 SD)	IUD (N=10): 50.7 ml (±34 SD)
**Krishna (1980)**	Intensity of menstrual flow	Self-recorded menstrual blood loss: whether more, less, or same as compared to pre-trial menstrual blood loss	*Aspirin*		*Placebo*			
			5/39 women ↑ menstrual blood loss vs pre-trial blood loss		6/39 women ↑ menstrual blood loss vs pre-trial blood loss			
**Pedron (1987)**	Menstrual blood loss volume	Alkaline hematin method	*Aspirin†*		*Pre-aspirin control cycle*			
			<60 mL (N=24): change in menstrual blood loss ranged from +51.4% up to +85%		<60 mL: 24 women			
			60-80 mL (N=16): change in menstrual blood loss ranged from -7.5% up to +22.4% (N=16)		60-80 mL: 16 women			

			≥80 mL (N=13): change in menstrual blood loss ranged from -17.6% up to +5.9%	≥80 mL: 13 women
**Wun (2013)**	Incidence of HMB	Menorrhagia not further specified<br><br>Either events requiring medical attention or recorded in retrospect during study visits by interviewing patients	*Prasugrel*	*Placebo*
			60 days incidence of menorrhagia in patients with SCD: 2/21 (9.5%)	60 days incidence of menorrhagia in patients with SCD: 0/9
**Cuadrado (2014)**	Incidence of HMB	Menorrhagia not further specified<br><br>Assessed by questionnaire at study visits or from general physician/hospital reports	*Aspirin*	*Aspirin + low intensity VKA‡*
			aPL positive patients, median FU 3 years: 0/80 reported menorrhagia	aPL positive patients, median FU 3 years: 10/80 (12.5%) reported menorrhagia
**Matyas (2015)**	Menstrual blood loss volume	Menstrual pictogram from Wyatt et al filled in by participants	Cycles with aspirin use (OTC, self-reported): mean 53.7 ml (±2.4SD) (N=39 cycles)	Cycles without aspirin use: mean 45.4 ml (±2.7SD) (N=470 cycles)
	Duration of menstrual bleeding	Menstrual pictogram from Wyatt et al filled in by participants	Cycles with aspirin use (OTC, self-reported): mean 7.9 days (±2.4SD) (N=39 cycles)	Cycles without aspirin use: mean 6.9 days (±2.2SD) (N=470 cycles)
	Intensity of menstrual flow	Menstrual pictogram from Wyatt et al filled in by participants	Cycles with aspirin use (OTC, self-reported): 42% heavy menstrual flow (N=39 cycles)	Cycles without aspirin use: 32% heavy menstrual flow (N=470 cycles)

<table>
<tr><td rowspan="3">Boonyawat (2021)</td><td rowspan="2">Duration of menstrual bleeding</td><td rowspan="2">Self-reported by participants, comparing menstrual flow duration of their last menstruation with their menstruation before the start of any anticoagulant therapy</td><td>Aspirin (N=108)§</td><td colspan="2">Rivaroxaban 20 mg (N=134)§</td><td colspan="2">Rivaroxaban 10 mg (N=120)§</td></tr>
<tr><td>9-12% ↑ duration</td><td colspan="2">12-18% ↑ duration</td><td colspan="2">6-12% ↑ duration</td></tr>
<tr><td>Intensity of menstrual flow</td><td>Self-reported by participants, comparing menstrual flow intensity of their last menstruation with their menstruation before the start of any anticoagulant therapy</td><td>13-20% ↑ flow intensity</td><td colspan="2">19-24% ↑ flow intensity</td><td colspan="2">14-21% ↑ flow intensity</td></tr>
<tr><td rowspan="2">Kawamatsu (2021)</td><td rowspan="2">Prevalence of HMB</td><td rowspan="2">Menorrhagia not further specified

Based on data collected from electronic medical records</td><td>Antiplatelet</td><td>DOAC</td><td colspan="2">VKA</td><td>Antiplatelet + anticoagulant</td></tr>
<tr><td>Patients with Fontan circulation: 2/28 (7.1%) menorrhagia</td><td>Patients with Fontan circulation: 1/16 (6.3%) menorrhagia</td><td colspan="2">Patients with Fontan circulation: 4/16 (25%) menorrhagia</td><td>Patients with Fontan circulation: 5/10 menorrhagia</td></tr>
<tr><td rowspan="2">Rodpetch (2021)</td><td rowspan="2">Duration of menstrual bleeding</td><td rowspan="2">Self-reported by participants, change in duration of menstrual bleeding before and after receiving oral antithrombotics</td><td>Antiplatelet (N=16)¶</td><td colspan="2">VKA (N=29)¶</td><td colspan="2">DOAC (N=4)¶</td></tr>
<tr><td>4.6 days (±1.9SD) before & after therapy**

12.5% ↑ duration</td><td colspan="2">4.5 (±2.0SD) before vs 5.2 (±2.4SD) after**

41.1% ↑ duration</td><td colspan="2">2.8 (±1.7SD) before vs 4 (±1.2SD) after**

50% ↑ duration</td></tr>
</table>

	Intensity of menstrual flow	Self-reported by participants, change in intensity of menstrual bleeding before and after receiving oral antithrombotics	37.5% ↑ flow intensity	65.5% ↑ flow intensity	75% ↑ flow intensity
	Prevalence of HMB	HMB defined as MBQ score of ≥21.5	HMB: 25.0% (95% CI, 7.0-52.0)	HMB: 27.6% (95% CI, 12.7-47.0)	HMB: 25.0% (95% CI, 0.6-80.0)
**Matsushita (2022)**	Prevalence of HMB	HMB defined as usual rate of changing pads during full flow higher than 8 times daily<br><br>Based on a structured questionnaire	*Antiplatelet*	*Anticoagulant*	*Combination*
			Women with Fontan circulation: 3/8 HMB	Women with Fontan circulation: 2/8 HMB	Women with Fontan circulation: 2/2 HMB

*Mean menstrual blood loss volume per menstrual cycle; †percentage change in menstrual blood loss volume from the pre-aspirin control cycle, over 4 consecutive menstrual cycles; ‡target international normalized ratio 1.5 (range 1.3-1.7); §menstrual flow duration and intensity versus prior any anticoagulant therapy, over 5 follow-up visits during the 1-year follow-up period; ¶self-reported increased menstrual flow duration and intensity versus prior antiplatelet or anticoagulant therapy; ** mean (±SD) duration of menstrual bleeding before and after antiplatelet or anticoagulant therapy.

Abbreviations: aPL = antiphospholipid antibodies; CI = confidence interval; DOAC = direct oral anticoagulant; FU = follow-up; HMB = heavy menstrual bleeding; IUD = intra-uterine device; MBL = menstrual blood loss; MBQ = menstrual bleeding questionnaire; OTC = over-the-counter; SCD = sickle cell disease; SD = standard devation; VKA = vitamin K antagonist.

Supplementary table S2. Study results on chronic antiplatelet therapy

Author (year)	Study design	Overall risk of bias*	Antiplatelet drug type & dose	Assessment method & definition	Intervention	Comparison		
Wun (2013)	Randomized double-blind controlled phase 2 trial	No information	Prasugrel 5 mg daily	Menorrhagia not further specified<br><br>Either events requiring medical attention or recorded in retrospect during study visits by interviewing patients	*Prasugrel*<br>60 days incidence of menorrhagia in patients with SCD: 2/21 (9.5%)	*Placebo*<br>60 days incidence of menorrhagia in patients with SCD: 0/9		
Cuadrado (2014)	Randomized open-label controlled trial	Serious	Aspirin 75-125 mg	Menorrhagia not further specified<br><br>Assessed by questionnaire at study visits or from general physician/hospital reports	*Aspirin*<br>aPL positive patients, median FU 3 years: 0/80 reported menorrhagia	*Aspirin + low intensity VKA†*<br>aPL positive patients, median FU 3 years: 10/80 (12.5%) reported menorrhagia		
Boonyawat (2021)	Parallel-group double-blind randomized trial	Moderate	Aspirin 100 mg daily	Self-reported by participants, comparing menstrual flow duration and intensity of their last menstruation with their menstruation before the start of any anticoagulant therapy	*Aspirin (N=108)‡*<br>9-12% ↑ duration<br><br>13-20% ↑ flow intensity	*Rivaroxaban 20 mg (N=134)‡*<br>12-18% ↑ duration<br><br>19-24% ↑ flow intensity	*Rivaroxaban 10 mg (N=120)‡*<br>6-12% ↑ duration<br><br>14-21% ↑ flow intensity	
Kawamatsu (2021)	Retrospective cohort study	Serious	Not reported	Menorrhagia not further specified	*Antiplatelet*	*DOAC*	*VKA*	*Antiplatelet + anticoagulant*

				Based on data collected from electronic medical records	Patients with Fontan circulation: 2/28 (7.1%) menorrhagia	Patients with Fontan circulation: 1/16 (6.3%) menorrhagia	Patients with Fontan circulation: 4/16 (25%) menorrhagia	Patients with Fontan circulation: 5/10 menorrhagia
Rodpetch (2021)	Cross-sectional study	Serious	Aspirin 81 mg, clopidogrel 75 mg	Self-reported by participants, change in duration and intensity of menstrual bleeding before and after receiving oral antithrombotics<br><br>HMB defined as MBQ score of ≥21.5	*Antiplatelet (N=16)§*	*VKA (N=29)§*	*DOAC (N=4)§*	
					12.5% ↑ duration<br><br>37.5% ↑ flow intensity<br><br>HMB: 25.0% (95% CI, 7.0-52.0)	41.1% ↑ duration<br><br>65.5% ↑ flow intensity<br><br>HMB: 27.6% (95% CI, 12.7-47.0)	50% ↑ duration<br><br>75% ↑ flow intensity<br><br>HMB: 25.0% (95% CI, 0.6-80.0)	
Matsushita (2022)	Prospective cohort study	Serious	Not reported	HMB defined as usual rate of changing pads during full flow higher than 8 times daily<br><br>Based on a structured questionnaire	*Antiplatelet*	*Anticoagulant*	*Combination*	
					Women with Fontan circulation: 3/8 HMB	Women with Fontan circulation: 2/8 HMB	Women with Fontan circulation: 2/2 HMB	

*Overall risk of bias judgements based on the Risk of Bias in Non-randomised Studies- of Interventions assessment tool; †target international normalized ratio 1.5 (range 1.3-1.7); ‡menstrual flow duration and intensity versus prior any anticoagulant therapy, over 5 follow-up visits during the 1-year follow-up period; §self-reported increased menstrual flow duration and intensity versus prior antiplatelet or anticoagulant therapy.

Abbreviations: aPL = antiphospholipid antibodies; CI = confidence interval; DOAC = direct oral anticoagulant; FU = follow-up; HMB = heavy menstrual bleeding; MBQ = menstrual bleeding questionnaire; SCD = sickle cell disease; VKA = vitamin K antagonist.

References

1 Aslonova IZ. Comparison Of Iron Deficiency Anemia Among Women. *NeuroQuantology*. 2022; **20**: 1246-9. 10.14704/nq.2022.20.8.NQ44137.

2 Dubrall D, Just KS, Schmid M, Stingl JC, Sachs B. Adverse drug reactions in older adults: A retrospective comparative analysis of spontaneous reports to the German Federal Institute for Drugs and Medical Devices. *BMC Pharmacol Toxicol*. 2020; **21**. 10.1186/s40360-020-0392-9.

3 Islam MA, Alam F, Gan SH, Sasongko TH, Wan Ghazali WS, Wong KK. A 15-year single centre retrospective study of antiphospholipid syndrome patients from Northern Malaysia. *Malays J Pathol*. 2017; **39**: 123-33.

4 Jimeno-Sanchez J, Fuertes-Ferre G, Ruiz-Aranjuelo A, Caballero-Jambrina I, Diarte-de Miguel JA, Ortas-Nadal MR. Sex-specific differences in antithrombotic therapy and prognosis in patients with acute coronary syndrome treated with stent. *Arch Cardiol Mex*. 2019; **89**: 330-8. 10.24875/acm.19000058.

5 Jukic AMZ, Padiyara P, Bracken MB, McConnaughey DR, Steiner AZ. Analgesic use at ovulation and implantation and human fertility. *Am J Obstet Gynecol*. 2020; **222**: 476.e1-.e11. 10.1016/j.ajog.2019.11.1251.

6 Levi M, Rosselli M, Simonetti M, Brignoli O, Cancian M, Masotti A, Pegoraro V, Cataldo N, Heiman F, Chelo M, Cricelli I, Cricelli C, Lapi F. Epidemiology of iron deficiency anaemia in four European countries: a population-based study in primary care. *Eur J Haematol*. 2016; **97**: 583-93. 10.1111/ejh.12776.

7 Levi M, Simonetti M, Marconi E, Brignoli O, Cancian M, Masotti A, Pegoraro V, Heiman F, Cricelli C, Lapi F. Gender differences in determinants of iron-deficiency anemia: a population-based study conducted in four European countries. *Ann Hematol*. 2019; **98**: 1573-82. 10.1007/s00277-019-03707-w.

8 Mauer AC, Khazanov NA, Levenkova N, Tian S, Barbour EM, Khalida C, Tobin JN, Coller BS. Impact of sex, age, race, ethnicity and aspirin use on bleeding symptoms in healthy adults. *J Thromb Haemost*. 2011; **9**: 100-8. 10.1111/j.1538-7836.2010.04105.x.

9 Moore N, Charlesworth A, Van Ganse E, LeParc JM, Jones JK, Wall R, Schneid H, Verrière F. Risk factors for adverse events in analgesic drug users: Results from the PAIN study. *Pharmacoepidemiol Drug Saf*. 2003; **12**: 601-10. 10.1002/pds.842.

10 Schindewolf M, Beyer-Westendorf J, Balradj J, Bowrin K, Huelsebeck M, Briere JB. Systematic Literature Review of Randomized Trials Comparing Antithrombotic Therapy Following Revascularization Procedures in Patients With Peripheral Artery Disease. *Angiology*. 2020; **71**: 773-90. 10.1177/0003319720936505.

11 Soe MZ, Lin CLS, Hayati F, Murugaiah C. Letter to: Successful management of acute heavy menstrual bleeding with Foley's catheter of a patient under combined antiplatelet therapy for cardiac disease: A case report. *Gazi Med J*. 2021; **32**: 348-9. 10.12996/gmj.2021.80.

12 Tsampras N, Ma K, Arora R, McLeod G, Minchelotti F, Craciunas L. Office hysteroscopy safety and feasibility in women receiving anticoagulation and anti-platelet treatment. *Eur J Obstet Gynecol Reprod Biol*. 2021; **260**: 110-3. 10.1016/j.ejogrb.2021.03.022.

13 Uchiyama S, Tanahashi N, Minematsu K. Clopidogrel two doses comparative 1-year assessment of safety and efficacy (COMPASS) study in japanese patients with ischemic stroke. *Cerebrovasc Dis*. 2012; **34**: 229-39. 10.1159/000342655.

14 Akiyama S, Tanaka E, Cristeau O, Onishi Y, Osuga Y. Treatment patterns and healthcare resource utilization and costs in heavy menstrual bleeding: a Japanese claims database analysis. *J Med Econ*. 2018; **21**: 853-60. 10.1080/13696998.2018.1478300.

15 Bloch B, Kort H. *Thrombocytopathia as a cause of menorrhagia: Two case reports*. Wiley Online Library, 1977.

16 Khalife R, Duffett L, Wang TF, Tinmouth A. *New onset of acute heavy menstrual bleeding in a 34-year-old woman*. Can Med Assoc, 2021.
17 Chae H, Park YK, Suh KP. A comparative study on anticoagulants following valve replacement surgery-a retrospective study with warfarin anticoagulation comparing with antiplatelet therapy in patients with bioprosthetic heart valve replacement. *Journal of Chest Surgery.* 1987; **20**: 13-21.
18 Arachchillage D, Cohen H. Recurrent Miscarriages and Neurological Symptoms. *Stroke Medicine*. 2015.
19 Gu ZC, Shi FH, Zhu J, Wan F, Shen L, Li H. The Management of Heavy Menstrual Bleeding After Percutaneous Coronary Intervention in a Woman of Reproductive Age. *Front Pharmacol*. 2018; **9**: 1573. 10.3389/fphar.2018.01573.
20 Knol HM, Bogchelman DH, Kluin-Nelemans HC. Routine evaluation and treatment of unexplained menorrhagia: do we consider haemostatic disorders? *European Journal of …*. 2010.
21 Matsumoto Y, Fukushima S, Shimahara Y, Tadokoro N, Kakuta T, Kobayashi J, Fujita T. Sex differences in continuous-flow ventricular assist device therapy for advanced heart failure. *Gen Thorac Cardiovasc Surg*. 2021; **69**: 919-25. 10.1007/s11748-020-01538-5.
22 Själander A, Friberg B, Svensson P, Stigendal L, Lethagen S. Menorrhagia and minor bleeding symptoms in women on oral anticoagulation. *J Thromb Trombolysis*. 2007; **24**: 39-41. 10.1007/s11239-006-0003-7.
23 Soni H, Kurkowski J, Guffey D, Dietrich JE, Srivaths LV. Gynecologic Bleeding Complications in Postmenarchal Female Adolescents Receiving Antithrombotic Medications. *J Pediatr Adolesc Gynecol*. 2018; **31**: 242-6. 10.1016/j.jpag.2017.12.007.
24 Nguyen JT, Koerper MA, Hess CP, Dowd CF, Hoffman WY, Dickman M, Frieden IJ. Aspirin therapy in venous malformation: A retrospective cohort study of benefits, side effects, and patient experiences. *Pediatr Dermatol*. 2014; **31**: 556-60. 10.1111/pde.12373.
25 Beelen P, van der Velde MGAM, Herman MC, Geomini PM, van den Brink MJ, Duijnhoven RG, Bongers MY. Treatment of women with heavy menstrual bleeding: Results of a prospective cohort study alongside a randomised controlled trial. *Eur J Obstet Gynecol Reprod Biol*. 2021; **257**: 1-5. 10.1016/j.ejogrb.2020.11.071.
26 Iyengar AJ, S.Winlaw D, Galati JC, Wheaton GR, Gentles TL, Grigg LE, Justo RN, Radford DJ, Attard C, G.Weintraub R, Bullock A, Sholler GS, Celermajer DS, d'Udekem Y. No difference between aspirin and warfarin after extracardiac Fontan in a propensity score analysis of 475 patients. *Eur J Cardio-thorac Surg*. 2016; **50**: 980-7. 10.1093/ejcts/ezw159.
27 von Beckerath O, Paulitschek AM, Kröger K, Kowall B, Santosa F, Stang A. Increasing use of anticoagulants in Germany and its impact on hospitalization rates for genitourinary bleeding. *J Thromb Thrombolysis*. 2020; **49**: 533-9. 10.1007/s11239-020-02061-3.
28 Borzutzky C, Jaffray J. Diagnosis and management of heavy menstrual bleeding and bleeding disorders in adolescents. *JAMA pediatrics*. 2020.
29 Fitzmaurice DA, Blann AD, Lip GYH. ABC of antithrombotic therapy: Bleeding risks of antithrombotic therapy. *Br Med J*. 2002; **325**: 828-31. 10.1136/bmj.325.7368.828.
30 Hayes SN. First, do no harm. *European Heart Journal*. 2021.
31 Hayes SN, Kim ESH, Saw J, Adlam D. Spontaneous coronary artery dissection: current state of the science: a scientific statement from the American Heart Association. *Circulation*. 2018.
32 James AH. Heavy menstrual bleeding: work-up and management. *Hematology Am Soc Hematol Educ Program.* 2016;**2016**:236-242.
33 James AH, Kouides PA, Abdul-Kadir R, Dietrich JE, Edlund M, Federici AB, Halimeh S, Kamphuisen PW, Lee CA, Martínez-Perez O, McLintock C, Peyvandi F, Philipp C, Wilkinson J, Winikoff R. Evaluation and management of acute menorrhagia in women with and without underlying bleeding disorders:

consensus from an international expert panel. *Eur J Obstet Gynecol Reprod Biol*. 2011;**158**:124-34. doi: 10.1016/j.ejogrb.2011.04.025.
34 Jayasinghe R, Markham R. Dual antiplatelet therapy: management in general practice. *Australian Family Physician*. 2013.
35 Lindley KJ, Bairey Merz CN, Davis MB, Madden T, Park K, Bello NA; American College of Cardiology Cardiovascular Disease in Women Committee and the Cardio-Obstetrics Work Group. Contraception and Reproductive Planning for Women With Cardiovascular Disease: JACC Focus Seminar 5/5. *J Am Coll Cardiol*. 2021;**77**:1823-1834. doi: 10.1016/j.jacc.2021.02.025.
36 Maas AH, Euler M, Bongers MY, Rolden HJ, Grutters JP, Ulrich L, Schenck-Gustafsson K. Practice points in gynecardiology: Abnormal uterine bleeding in premenopausal women taking oral anticoagulant or antiplatelet therapy. *Maturitas*. 2015; **82**: 355-9. 10.1016/j.maturitas.2015.08.014.
37 Rivara A, James AH. Managing Heavy Menstrual Bleeding in Women at Risk of Thrombosis. *Clin Obstet Gynecol*. 2018; **61**: 250-9. 10.1097/grf.0000000000000372.
38 Cosyns S, Dony N, Polyzos NP. Can ulipristal acetate be a treatment option for patients with menorrhagia and a history of ischemic cerebrovascular accident? *J Obstet Gynaecol*. 2016; **36**: 859-60. 10.1080/01443615.2016.1174832.
39 Soykan Y, Demirdağ E, Nas T. Successful management of acute heavy menstrual bleeding with foley's catheter of a patient under combined antiplatelet therapy for cardiac disease: A case report. *Gazi Med J*. 2020; **31**: 683-5. 10.12996/gmj.2020.159.
40 Lukes AS, Reardon B, Arepally G. Use of the levonorgestrel-releasing intrauterine system in women with hemostatic disorders. *Fertil Steril*. 2008; **90**: 673-7. 10.1016/j.fertnstert.2007.07.1315.
41 Bala MM, Paszek E, Lesniak W, Wloch-Kopec D, Jasinska K, Undas A. Antiplatelet and anticoagulant agents for primary prevention of thrombosis in individuals with antiphospholipid antibodies. *Cochrane Database Syst Rev*. 2018; **7**: CD012534. 10.1002/14651858.CD012534.pub2.
42 Godfrey EM, Folger SG, Jeng G, Jamieson DJ, Curtis KM. Treatment of bleeding irregularities in women with copper-containing IUDs: A systematic review. *Contraception*. 2013; **87**: 549-66. 10.1016/j.contraception.2012.09.006.
